# Evolution and Neural Network Prediction of CO_2_ Emissions in Weaned Piglet Farms

**DOI:** 10.3390/s22082910

**Published:** 2022-04-11

**Authors:** Manuel R. Rodriguez, Roberto Besteiro, Juan A. Ortega, Maria D. Fernandez, Tamara Arango

**Affiliations:** 1 Department of Agroforestry Engineering, Higher Polytechnic Engineering School, University of Santiago de Compostela, 27002 Lugo, Spain; mdolores.fernandez@usc.es (M.D.F.); tamara.arango@rai.usc.es (T.A.); 2 Centro de Investigaciones Agrarias de Mabegondo, Xunta de Galicia, 15318 A Coruña, Spain; roberto.besteiro.doval@xunta.es; 3 Consellería do Medio Rural, Xunta de Galicia, 36500 Lalin, Spain; juan.antonio.ortega.martinez@xunta.es

**Keywords:** CO_2_ concentration, CO_2_ emission, neural network, weaned piglets, post weaning cycle

## Abstract

This paper aims to study the evolution of CO_2_ concentrations and emissions on a conventional farm with weaned piglets between 6.9 and 17.0 kg live weight based on setpoint temperature, outdoor temperature, and ventilation flow. The experimental trial was conducted during one transition cycle. Generally, the ventilation flow increased with the reduction in setpoint temperature throughout the cycle, which caused a reduction in CO_2_ concentration and an increase in emissions. The mean CO_2_ concentration was 3.12 g m^–3^. Emissions of CO_2_ had a mean value of 2.21 mg s^−1^ per animal, which is equivalent to 0.195 mg s^−1^ kg^−1^. A potential function was used to describe the interaction between 10 min values of ventilation flow and CO_2_ concentrations, whereas a linear function was used to describe the interaction between 10 min values of ventilation flow and CO_2_ emissions, with *r* values of 0.82 and 0.85, respectively. Using such equations allowed for simple and direct quantification of emissions. Furthermore, two prediction models for CO_2_ emissions were developed using two neural networks (for 10 min and 60 min predictions), which reached *r* values of 0.63 and 0.56. These results are limited mainly by the size of the training period, as well as by the differences between the behavior of the series in the training stage and the testing stage.

## 1. Introduction

Animal production is a fundamental activity for meeting the increasing global demand for food. However, this activity is a key contributor to global GHG emissions [1,2] and to the global problem of climate change [3]. Pig production accounts for a significant portion of these emissions [4], with a contribution of between 9 and 13% [2,3]. Considering the GHG produced by pigs and manure at pig house level, the gas that contributes the most to the greenhouse effect is CO_2_, with approximately 81% of emissions [5]. However, direct CO_2_ emissions, arising from the animals and manure, are generally excluded from GHG evaluation, because they are supposed to be compensated by CO_2_ consumption through photosynthesis, performed by the plants used as feed. For this reason, CO_2_ production is rarely addressed in the literature [5].

CO_2_ emissions from farms depend on two factors, CO_2_ production and ventilation rate. Here, the main source of CO_2_ is animal metabolism, followed by manure [6], although other sources may exist, such as heaters using propane or natural gas [7]. More specifically, the carbon dioxide produced by animals depends on animal weight and daily gain, while CO_2_ emissions are also strongly affected by variations in animal activity [8,9,10,11,12]. Carbon dioxide emissions have also been reported to be affected by other factors, such as diet composition [13,14] and conversion efficiency, droppings management, or environmental conditions [15], following a sinusoidal pattern of daily emission [8,9]. Nevertheless, there are some discrepancies between the results that have been reported so far, probably due to variations in animal species, feeding level, farm practices, control strategy, manure management systems, animal density, geography, climate, season, and other factors [7,10]. In relation to pig production, variations have been found between physiological stages [5,16], which could be attributed to the conditions of the building, ventilation system, management, diet, or even gas measurement method. Thus, while different authors [17,18] have reported fairly similar CO_2_ emissions during the fattening period, larger variations have been found during the other physiological stages [19,20,21], especially during weaning [5]. In fact, CO_2_ production per unit of total heat production is much lower for weaning pigs than for growing finishing pigs [12]. In addition, large variations have been found in GHG emissions per unit of product across EU countries, mainly because of the differences observed in the production systems [5], feed type, and efficiency of nutrient use [22].

Farm buildings for piglets in transition, where weaned piglets are housed from weaning up to fattening, as well as delivery rooms, are the facilities with the highest levels of CO_2_ concentration. This is because ventilation rates are kept reduced at this stage owing to the fact that animals require high temperatures and are very sensitive to air currents [23]. Consequently, although CO_2_ emissions are affected considerably more by ventilation rate than by indoor air concentration [24], these farms present high levels of CO_2_ emissions into the atmosphere due to the relationship between concentration and emission through the ventilation rate [24,25]. Moreover, the ventilation system and the control strategy can significantly affect the airflow inside livestock rooms, which can further affect the emission of gaseous contaminants from animal buildings [6].

Intelligent livestock farming, with a continuous improvement in the production efficiency of management systems, can contribute to decreasing GHG emissions [26,27]. In recent years, technological progress has contributed to obtaining positive results in the reduction of GHG emissions from pig production [28], although other studies have shown that these efforts may not be enough to achieve a sustainable level [29,30]. Therefore, there is a need to find housing systems that minimize ammonia and GHG emissions, and improve animal welfare [31]. In this respect, most existing studies focus on GHG emissions for ruminants, partly because of their high emission per unit of production. In contrast, fewer studies focus on monogastric animals, like pigs [32,33]. New research is thus necessary in order to obtain accurate emission factors [4,10,15], mainly under farm conditions [34], that cover the full production seasons [14].

A number of studies have been performed to evaluate emissions from farms with weaned piglets, both in field conditions [14] and in experimental rooms, during part of the transition period [3,13]. Two of those studies focused on determining the influence of different diets on GHG emissions and found that including dried distillers grains with solubles in the diet of piglets did not cause significant differences in CO_2_ emissions [3]. In addition, using five different isoenergetic diets (control, low protein, inclusion of sugar beet pulp, addition of benzoic acid, and a combination of previous diets) only caused a 15% decrease in CO_2_ emissions for the combined diet [13]. Different floor systems (straw-based deep litter, sawdust-based deep litter, and fully slatted floors) were also analyzed, but no significant differences in CO_2_ emissions were found between systems [3]. There is a lack of studies aimed at quantifying CO_2_ emissions on farms raising weaned piglets based on continuous measurements performed under field conditions. In contrast to the above studies, in this paper, we analyze the data for a complete production cycle, from weaning to fattening, from a conventional farm. Continuous measurements of environmental parameters and ventilation flow have been used, which results in quality data for a largely unexplored production stage.

In addition to quantifying emissions, generating models that are capable of predicting the emission of gases from farms is necessary to achieve emission control through intelligent livestock farming. One of the emerging technologies for modelling and predicting farm emissions is artificial intelligence (AI), which has already been used for developing many applications and papers in the field of smart farming. The most common AI models used for the modelling, prediction, and management of variables related to livestock farming are artificial neural networks (ANN), convolutional neural networks, deep learning, adaptive neural fuzzy inference system (ANFIS), machine learning, and pattern recognition [35]. Specifically, ANFIS models have been used for modelling ammonia emissions in pig farms [36], and ANN and ANFIS (artificial neural network and adaptive neuro-fuzzy inference systems, respectively) have been used for modelling and predicting energy output and greenhouse gas emissions from calf fattening farms [37].

This paper aims to quantify and study the evolution of CO_2_ concentration and emissions on a pig farm with weaned piglets from 6.9 to 17 kg LW during a complete transition cycle under field conditions. Our aim is to find the relationship between CO_2_ concentration and emissions, and some control parameters of the air conditioning system, such as setpoint temperature, outdoor temperature, or ventilation flow, during one transition cycle. In addition, we present a neural network-based model for the prediction of 10 min and 60 min CO_2_ emissions that can be incorporated into the environmental control system.

## 2. Materials and Methods

**Figure 1:**
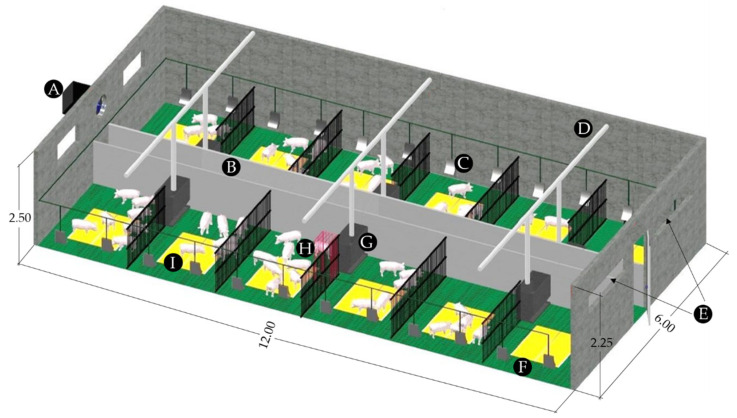
Overview of the room and location of the sensors: extractor fan (**A**), internal corridor (**B**), drinker (**C**), feed distributor (**D**), air inlet (**E**), slat (**F**), feeder (**G**), indoor sensor structure (**H**), and heating plate (**I**). The sensors for the speed of the air extracted through the ventilation system (*V_m_*) and CO_2_ concentration at the ventilation air outlet (*C_OUTLET_*) were located at (**A**) and the sensor for CO_2_ concentration in the exterior corridor of air inlet (*C_INLET_*) was located at (**E**).

Research was conducted in a conventional farm for the production of piglets located in NW Spain. Measurements were performed in a transition room, where piglets entered with an average weight of 6.9 kg at day 28 after birth and exited with 17.0 kg LW at day 65 after birth, covering a complete transition cycle, from 27 February to 4 April. An intermediate room was chosen in order to avoid the boundary effect of lateral positions in the building. The inner dimensions of the room, with polypropylene slats on the floor, were 11.82 m long by 5.86 m wide, and between 2.25 and 2.50 m high. The mean depth of the pit was 0.45 m. At the end of each cycle, the room was completely cleaned and manure was completely removed from the pit. The room had six pens on both sides of a central corridor and housed a maximum of 300 weaned piglets, with 50 piglets per pen, with 0.20 m^2^ per piglet (Figure 1).

The air conditioning system comprised ventilation and warm water underfloor heating facilities. Environmental control was performed in the building through a temperature probe, without altering the usual conditions of the farm. The setpoint temperatures (*T_S_*) defined for environmental control were between 26 and 23 °C with a bandwidth of ±1.5 °C, and decreased gradually with the increase in the age and weight of the animals (Table 1). Clean air entered the room through two windows with air deflectors on the wall opposite to the fan (Figure 1E). The animals were provided feed ad libitum through a screw conveyor system with a feeder shared by two pens. Water was supplied through two drinking cups in each pen.

The number of animals was registered daily. Animals were weighed at the beginning of the cycle, on day 28 after birth, on day 38 when the feed type was changed, and at the end of the cycle on day 65 after birth. In order to obtain the value for emissions per kg throughout the cycle, a linear interpolation was performed between the weighed values. Table 1 shows the mean weight of the piglets for each setpoint temperature.

Table 2 shows the environmental variables measured and the sensors used to measure them. Variables were measured every second, and the averages for every 10 min interval were stored in a HOBO^®^ data logger (Bourne, MA, USA).

Based on the speed of the air extracted, the flow extracted through the fan (*Q*) was calculated using the following expression:

*Q* = 1.143 *V_m_*·*S* (1)

where:

*Q* = flow extracted through the fan (m^3^ s^−1^);
*V_m_* = mean speed (m s^−1^);
1.143 = relation between *V_m_* at every measured point and speed measured at the location of the sensor, in m s^−1^;
*S* = duct section (0.303 m^2^).

The emission of CO_2_ per animal, *E*_CO2_, was calculated according to the following expression:

*E*_CO2_ = (*C_OUTLET_* − *C_INLET_*) *Q*/*n* (2)

where:

*E*_CO2_*=* emission of CO_2_ per animal (mg s^−1^);
*C_OUTLET_* = concentration of CO_2_ at the ventilation air outlet (mg m^−3^), measured at a height of 2.30 m;
*C_INLET_* = concentration of CO_2_ in the exterior corridor of air inlet (mg m^−3^), obtained as a mean of the measurements performed at four positions at an average temperature of 17.75 °C;
*n* = number of animals in the room.

### 2.1. Prediction of CO_2_ Emissions

Neural networks were trained to predict CO_2_ emissions at 10 min and 60 min intervals. An artificial neural network (ANN) is a method used for modelling non-linear relationships between input and output variables [38]. An ANN does not require previous knowledge about how to handle the system, which turns it into a very efficient and versatile technique in complex processes. Since the 2000s, ANNs have been the most recurring artificial intelligence models in the field of research on animal farming, mainly for detecting illnesses, estimating growth performance, or monitoring and controlling environmental conditions [35]. Since then, different training algorithms have been used, such as back propagation, radial basis function, or feed forward [37,39]. In this paper, a multilayer perceptron neural network was used. A resilient backpropagation method with weight backtracking, available in the “neuralnet” pack of R [40], was used to train the ANN. This algorithm is a first-rate optimization method similar to the backpropagation algorithm, but is generally faster, without the requirement of specifying free parameter values [41].

The number of neurons in the input and output layers is determined by the input and output variables of the model. In this case, three inputs were used, namely the values of *Q*, *T_IN_*, and *C*_CO2_ measured during the previous 10 min or 60 min periods, to obtain the output, *E*_CO2_. On the contrary, there is no specific procedure to determine the number of neurons, M, of the hidden layer. This value is defined through a procedure of trial and error. After having checked the performance of different neural network structures, and having evaluated the average degree of fit of up to 40 training iterations with each structure, we chose a network with an input layer of three neurons and one output layer of one neuron, with two hidden layers, and a total of 3-4-4-1 neurons per layer. This structure was the most efficient at predicting emissions for both the 10 min and 60 min periods. Moreover, the initial weights of the network were allocated randomly. A logistic activation function was used because of the common use of these functions in the field of agriculture [37]. The stopping criterion was set to 0.01, with a maximum number of steps for the training of 1 × 10^5^.

### 2.2. Evaluation of the Performance of the Model

In order to evaluate the performance of the model, three statistical measures were used: root mean square error (*RMSE*), Pearson correlation coefficient (*r*), and mean absolute relative error (*MARE*). *RMSE* was used to measure the difference between the predictions of the model and the observed values. The values of *RMSE* are expressed in units, while *MARE* indicates the size of the error in relative terms. The Pearson correlation coefficient indicates the strength and direction of a linear relationship between the predicted and observed values.

(3) $$RMSE=\sqrt{\frac{\sum_{i=1}^{n}{\left(y-y^{\prime }\right)}^{2}}{n}}$$

(4) $$MARE=\frac{1}{n}\;\overset{n}{\underset{i=1}{\sum }}\left|\frac{y^{\prime }-y}{y}\right|$$

(5) $$r=\frac{\sum_{i=1}^{n}\left(y-\bar{y}\right)\left(y^{\prime }-{\bar{y}}^{’}\right)}{\sqrt{\sum_{i=1}^{n}{\left(y-\bar{y}\right)}^{2}}\sqrt{\sum_{i=1}^{n}{\left(y^{\prime }-{\bar{y}}^{’}\right)}^{2}}}$$

where y is the observed value; $y^{\prime }$ is the predicted value; $\bar{y}$ and ${\bar{y}}^{’}$ are the average values of y and $y^{\prime }$, respectively; and *n* is the number of values.

## 3. Results and Discussion

The mean CO_2_ concentration at the air outlet of the building where piglets between 6.9 kg and 17.0 kg were housed was 1852 ± 711 ppm, which is equivalent to 3.12 ± 1.20 g m^–3^ (Table 3). These values were near the lowest mean value of 1981–3253 ppm found in different trials for pigs between 5.5 and 118 kg, with highly extreme temperatures and very restricted ventilation over a prolonged period in the cycle [14], and slightly higher than the mean of 2.89 g m^–3^ found for pigs between 13 and 32 kg in environmental chambers [13]. All of these CO_2_ concentrations were far below the 5.30 mg m^−3^ obtained for animals between 17 and 20 kg [20].

The mean CO_2_ emission was 2.21 ± 1.09 mg s^−1^ pig^−1^ (Table 4), which is below the emissions reported by other authors in buildings with fully slatted floors [3] for pigs in the same growth stage, with very similar weights of 7 and 23 kg and emission values of 3.51 and 3.95 mg s^−1^ pig^−1^. By transforming the unit based on animal weight, the emission obtained was 0.195 ± 0.074 mg s^−1^ kg^−1^. This value was only above the 0.139 mg s^−1^ kg^−1^ found for pigs of 7 to 35 kg [19], and well below the values reported in other studies that analyzed different diets, with mean values of 0.293 mg s^−1^ kg^−1^ for piglets between 13 and 32 kg [13], and values of 0.423 mg s^−1^ kg^−1^ and 0.549 mg s^−1^ kg^−1^ for pigs between 5.5 and 118 kg [14]. Even though both studies focused on pigs with weights within the range analyzed in our study, the study in [13] started at much heavier weights than the weights studied in our paper and ended much later, while the study in [14] started at a slightly lower weight, but included the finishing stage, so that the animals ended up being much older. The weaned piglet stage is the one with the largest variations in CO_2_ emissions, whereas other stages produce more homogenous emissions [5]. Despite this, the studies devoted to the effect of different diets on weaned piglets have not found significant differences on CO_2_ emissions [13,14]. Likewise, using a fully slatted floor as opposed to straw-based deep litter or sawdust-based deep litter did not cause significant differences in CO_2_ emissions [3].

### 3.1. Variation of CO_2_ Concentration and Emissions with Setpoint Temperature

**Figure 2:**
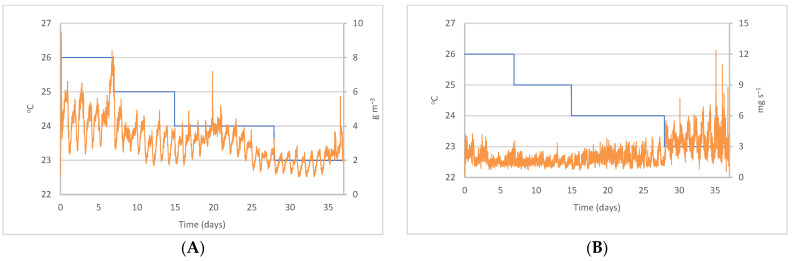
Evolution during the cycle of (**A**) CO_2_ concentration, – *C*_CO2_ (g m^−3^), and (**B**) CO_2_ emissions per animal, – *E*_CO2_ (mg s^−1^), with setpoint temperature, – *T_S_* (°C).

The CO_2_ concentration in the building reached its peak with the highest setpoint temperature, 26 °C, set at the start of the cycle, and decreased as animals grew and the setpoint temperature was reduced (Table 3, Figure 2A). The mean CO_2_ concentrations for every setpoint temperature were lower than the measurements reported by [42], although concentrations were always in the range reported by a number of authors for pig farms [43,44,45]. In summary, the CO_2_ concentration declined as the animals grew. In contrast, another study reported increasing concentrations [13], which may be caused by the different ages of the animals (28 days vs. 37 days). As a result of age difference, higher temperatures and lower ventilation rates were required in our study to ensure animal welfare conditions. Actually, a trend of increasing concentrations was observed in the final stage of the cycle analyzed in our study (Figure 2A).

CO_2_ emissions decreased at the beginning of the cycle because of the limitation of ventilation flows and the weight of piglets (Table 4 and Figure 2B). Emissions during the first five days (*T_S_* = 26 °C) were higher than on the following days (change to *T_S_* = 25 °C) because the piglets showed more activity as a consequence of post-weaning stress. From that moment, emissions increased with the age of the animals, which was particularly noticeable at the end of the cycle (from day 28), as stated by [2]. The regular increase in CO_2_ emissions over the cycle suggests the influence of animal weight [3], which results from an increase in the metabolic rate and in CO_2_ production due to breathing [14].

### 3.2. Variation of CO_2_ Concentrations and Emission with Outdoor Temperature

**Figure 3:**
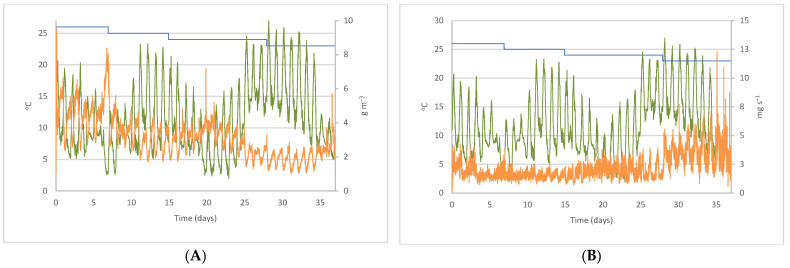
Evolution throughout the cycle of outdoor temperature, – *T_OUT_* (°C) and setpoint temperature – *T_S_* (°C) with (**A**) CO_2_ concentration, – *C*_CO2_ (g m^−3^), and (**B**) CO_2_ emissions per animal, – *E*_CO2_ (mg s^−1^).

The daily behavior of the CO_2_ concentration was inverse to the evolution of the outdoor temperature (Figure 3A), with the highest concentrations occurring at night. This resulted in a negative correlation, with a value of *r* = −0.695 for the cycle, which was above the value obtained by Ni et al. [46] for two manure-belt layer hen houses.

As shown in Figure 3B, during the first three days of the cycle, with *T_S_* = 26 °C, the outdoor temperature and CO_2_ emissions followed a similar pattern because of the effects of outdoor temperature on the ventilation system. On the following days, CO_2_ emissions decreased steadily until day 25, when outdoor temperature started to increase. During this interval, both variables showed a weak, inverse correlation (*r* = −0.475), which was in agreement with the correlation found by Ni et al. [46] for laying hens. This means that minimum emissions occurred at maximum outdoor temperatures. From that day until the end of the cycle, emissions followed a daily oscillation that was parallel to that of outdoor temperatures.

### 3.3. Variation of CO_2_ Concentrations and Emissions with Ventilation Flow

**Figure 4:**
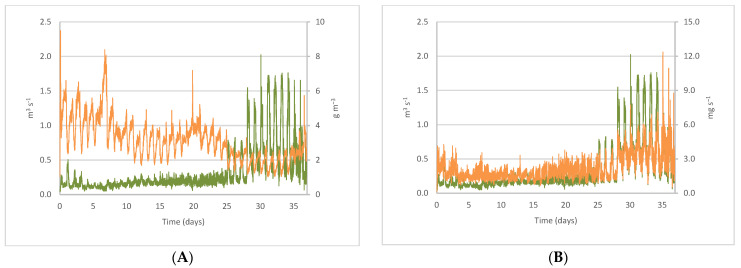
Evolution of ventilation flow, – *Q* (m^3^ s^−1^) during the cycle, with (**A**) concentration of CO_2_, – *C*_CO2_ (g m^−3^), and (**B**) emission of CO_2_ per animal, – *E*_CO2_ (mg s^−1^).

The ventilation flow was highly reduced during the first days of the cycle, until day 25, with a mean of 0.17 m^3^ s^−1^ and a maximum of 0.50 m^3^ s^−1^ (Figure 4). After that, a transition stage began for three days with a mean of 0.33 m^3^ s^−1^ and a maximum of 0.82 m^3^ s^−1^. From day 28, ventilation flow was significantly increased, with a mean of 0.74 m^3^ s^−1^ and a peak of 2.02 m^3^ s^−1^. Consequently, CO_2_ concentrations varied depending on the ventilation flow, such that, as the flow increased, concentration decreased both throughout the cycle [13] and on a daily basis [6]. Maximum CO_2_ concentrations (Figure 4A) of 9.50 g m^−3^ were measured during the first 25 days, when the highest mean value of 3.63 g m^−3^ was reached. This means that, as stated by [23] and contrary to what is usually anticipated, maximum CO_2_ concentrations occurred when the animals were younger. Furthermore, as the ventilation flow increased, the CO_2_ concentration decreased near the fan, and, therefore, in the building [24,25,47]. Thus, higher ventilation rates involved lower CO_2_ concentrations inside the facilities, albeit with higher emission rates [6].

**Figure 5:**
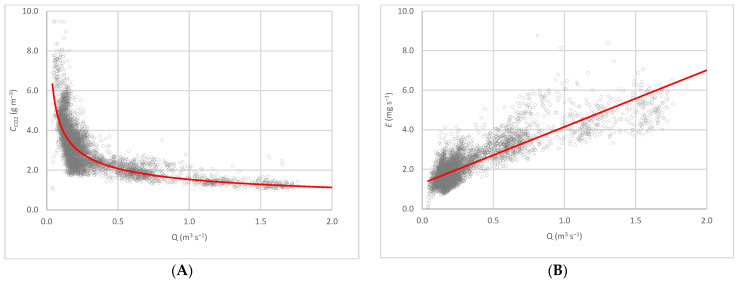
Correlation between (**A**) CO_2_ concentration, *C*_CO2_ (g m^−3^), **–** potential approximation, and (**B**) CO_2_ emission per animal, *E*_CO2_ (mg s^−1^), **–** linear relationship, and ventilation flow, *Q* (m^3^ s^−1^) ◦ measured values throughout the cycle.

The relationship between CO_2_ concentration (*C*_CO2_ in g m^−3^) and the flow of air extracted through the fan (*Q* in m^3^ s^−1^) during the cycle (Figure 5A) is given by the following equation:

*C*_CO2_ = 1.5316 *Q*^−0.442^ (6)

The correlation coefficient between the CO_2_ concentration and ventilation flow is *r* = 0.82.

CO_2_ emissions (Figure 4B) increased with the age of the piglets, in agreement with Stin et al. [16]. Mean CO_2_ emissions of 2.21 mg s^−1^ per animal were found for piglets with a mean weight of 11.39 kg during the cycle, although there was a sharp increase from day 28, with a peak of 12.34 mg s^−1^. Throughout the cycle, increasing the ventilation flow caused a rise in emissions, as observed for the fattening pigs [6,7]. As a consequence, CO_2_ emissions (*E*_CO2_ in mg s^−1^) and the flow of air extracted through the fan (*Q* in m^3^ s^−1^) showed a linear relationship (Figure 5B), which is given by the following equation:

*E*_CO2_ = 2.8624 *Q* + 1.2919 (7)

The correlation coefficient between CO_2_ emissions and ventilation flow is *r* = 0.85.

### 3.4. Neural Networks for the Prediction of CO_2_ Emissions

Table 5 shows the mean performance of the 10 min and 60 min prediction models after 40 iterations.

**Figure 6:**
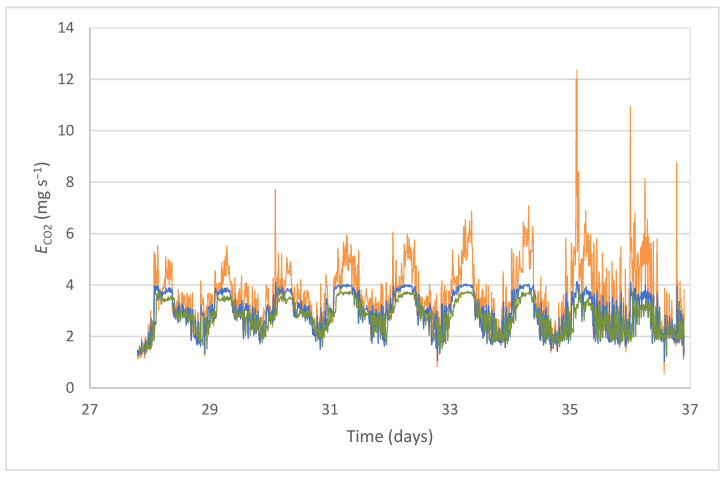
Measured (−) and predicted values of CO_2_ emission per animal, for a 10 min period (−) and a 60 min period (−), during the trial stage.

Expectedly, the 10 min prediction was more accurate than the hourly prediction, although statistical differences were minimal. In particular, *r* values of 0.63 and 0.56 were obtained with the 10 min and 60 min prediction models, respectively, whereas the *RMSE* values were 1.21 and 1.27 mg s^−1^, respectively. The prediction error of the models with regard to measured data in absolute terms (*RMSE*) was associated with very similar relative values (*MARE*), namely 0.23 for the 60 min prediction and 0.25 for the 10 min prediction. Figure 6 shows these differences. Furthermore, the hourly prediction model showed greater difficulty in predicting the peaks of CO_2_ emission.

The prediction results were limited mainly by the size of the training period and the differences between the behavior of the series in the training stage and the testing stage. As shown in Figure 6, the period for the data chosen as the training data corresponded to a stage of certain stability in emission values, while the emissions showed greater daily oscillations during the testing stage. These differences in the series limited the performance of the model and could account for its poor performance compared to the model proposed in [48], which returned an *r* value near 0.99 using a multilayer perceptron neural network. However, other authors [37] obtained an *r*^2^ of 0.733 and a *MARE* of 0.025 using an ANN prediction model of GHG emissions in a farm for calves. Xie et al., 2017 [36], used an ANFIS model to predict ammonia emissions in a fattening room for pigs and found an *r*^2^ of 0.635 and 0.648, with a *MARE* of 0.312 and 0.237, in contrast to the values of 0.23 and 0.25 reported above. In the discussed cases, the available dataset was larger than the one used in this study. Self-learning in neural networks with a larger dataset would lead to a better performance of the models, particularly because two of the input variables (ventilation flow and CO_2_ concentration) were key parameters in the emissions.

### 3.5. Innovative Potential of the Results

This paper focuses on CO_2_ concentrations and emissions in pig farms during weaning, the production stage with the largest variations in values obtained for emissions [5]. Such variations may be related to the complexity of the farm climate control system, which has to meet the strict environmental requirements of piglets. Furthermore, these requirements change considerably throughout the cycle. For this reason, conventional farms usually have air conditioning systems that include heating and forced ventilation. These were the conditions for this research, performed in a conventional farm, during the complete transition cycle, from the weaning of piglets to their transfer to fattening. New values were provided for this stage, which requires deeper study in terms of the quantification of GHG concentrations and emissions [15,34].

CO_2_ is the gas that contributes the most to the global emissions of greenhouse gases in pig production, accounting for approximately 81% [5]. Therefore, estimating CO_2_ emissions provides highly relevant information for global estimations. CO_2_ concentrations can be measured affordably, reliably, and with little maintenance using infrared sensors, which can be implemented on farms. Yet, the large number of rooms in transition farms would substantially increase the costs [49]. Moreover, for the estimation of emissions, the flow of the ventilation system must be determined. Ventilation flow may be obtained using the values provided by the controller, which can be easily measured, along with the fan curves or through previous calibrations using an anemometer.

This study evidenced the relationship between CO_2_ concentrations and emissions with the different operating parameters of the climate control system, namely setpoint temperature or ventilation flow, and with outdoor temperature, which directly influences climate control. By using these parameters, the CO_2_ concentrations and emissions can be estimated for any farm at any given moment, because 10 min values are used. In addition, we proposed Equations (6) and (7), which allow for a simple and direct estimation of the CO_2_ concentrations and emissions with high correlation indexes. Furthermore, because CO_2_ concentrations and emissions are linked to farm control parameters, emissions can also be regulated by operating the climate control system.

Finally, generating neural network-based models for 10 min and 60 min CO_2_ emissions allows for the prediction of the evolution of the study variables based on the input variables, namely flow extracted through the fan, temperature inside the building, and CO_2_ concentration, which provides a deeper understanding of the problem and the possibility to regulate emissions. In fact, good results have been obtained in studies focused on the optimization of environmental conditions in buildings based on machine learning systems [50]. These predictive systems can be incorporated into climate control systems to automatically optimize concentrations, consequently improving animal welfare, and emissions, consequently improving the environment.

In addition, an optimizer could be designed to simultaneously achieve a suitable distribution of temperatures and pollutant concentrations, yet using another model to predict indoor concentrations.

## 4. Conclusions

The following conclusions can be drawn from the analysis of variations in CO_2_ concentration and emissions in a conventional farm building with a fully slatted floor and mechanical ventilation for weaned piglets from 6.9 to 17.0 kg LW, and the relationship of such variations with setpoint temperature, outdoor temperature, and ventilation flow during a complete production cycle:

1. Generally, the ventilation flow increased with the reduction in setpoint temperature as the animals grew, which caused a reduction in CO_2_ concentration in the building. The mean value of the CO_2_ concentration was 3.12 g m^–3^. Maximum concentrations were obtained during the first days of the cycle, when ventilation was restricted due to the demanding thermal requirements of the piglets and their susceptibility to air currents.
2. CO_2_ emissions increased throughout the cycle. The emission pattern matched the pattern of outdoor temperatures when the ventilation flow was low and setpoint temperatures were high, at the start of the cycle. On the contrary, with higher ventilation flows and lower setpoint temperatures, the daily evolution of CO_2_ emissions was inverse to the evolution of the outdoor temperature. Emissions of CO_2_ showed a mean of 2.21 mg s^−1^ per animal, which was equivalent to 0.195 mg s^−1^ kg^−1^, with a total emission per animal of 7.05 kg at the end of the cycle.
3. Mathematical expressions for 10 min values of CO_2_ concentrations and emissions were obtained based on ventilation flow, a variable controlled by the climate control system, with *r* values of 0.82 and 0.85, respectively, which allowed for simple and direct quantification.
4. Using two neural networks, the 10 min and 60 min prediction models for CO_2_ emissions reached *r* values of 0.63 and 0.56. These results were mainly limited by the size of the training period, as well as by the differences between the behavior of the series in the training stage and the testing stage.
5. Both the equations and the ANN models for farm emissions allowed for the prediction of the values of emissions from the variables involved in environmental control. Therefore, the proposed equations and models could be integrated into climate control systems, thus allowing for emission regulation.

## Figures and Tables

**Table 1:** Setpoint temperature (*T_S_*), day of the cycle on which *T_S_* was set, piglet age, mean weight of piglets, and number of data.

*T_S_* (°C)	Setting of *T_S_* (Day of the Cycle)	Piglet Age (Days)	Mean Weight of Piglets (kg)	Number of Data
26	1	28	7.46	994
25	8	36	8.90	1152
24	16	44	12.12	1872
23	29	57	15.58	1297
CYCLE			11.39	5315

**Table 2:** Variables measured and sensors used in the experimental trial.

Variable	Name	Sensor	Measurement Range	Accuracy
CO_2_ concentration at the ventilation air outlet	*C_OUTLET_*	Transmitter (Delta Ohm HD37BTV.1, Delta Ohm, Caselle di Selvazzano, Italy)	0–5000 ppm	50 ppm ± 4%
CO_2_ concentration in the exterior corridor of air inlet	*C_INLET_*			
Outdoor temperature on the farm plot	*T_OUT_*	S-THB-M002 sensor installed in an EIC Control U-30 weather station (Onset Computer Corp., Bourne, MA, USA)	−40–75 °C	±0.2 °C from 0 °C to 50 °C
Indoor temperature	*T_IN_*			
Speed of the air extracted through the ventilation system	*V_m_*	Active air speed transmitter (Delta Ohm HD2903TTC310, Delta Ohm, Caselle di Selvazzano, Italy)	0.20–20 m s^−1^	±0.4 m s^−1^ + 3% of measurement

**Table 3:** Main statistics for CO_2_ concentration in the building for different setpoint temperatures.

*T_S_*	Maximum	Minimum	Mean	SDE
(°C)	(g m^3^)			
26	9.50	1.05	4.59	1.22
25	8.07	1.69	3.34	0.89
24	7.19	1.31	2.97	0.74
23	5.69	1.02	2.02	0.56
CYCLE	9.50	1.02	3.12	1.20

**Table 4:** Main statistics for CO_2_ emissions from the building per animal and per weight for different setpoint temperatures.

*T_S_* (°C)	Emission	Maximum	Minimum	Mean	SDE
26	per animal (mg s^−1^)	4.262	0.103	1.842	0.600
	per weight (mg s^−1^ kg^−1^)	0.602	0.015	0.249	0.088
25	per animal (mg s^−1^)	3.554	0.774	1.548	0.341
	per weight (mg s^−1^ kg^−1^)	0.443	0.086	0.175	0.041
24	per animal (mg s^−1^)	3.926	0.699	1.875	0.578
	per weight (mg s^−1^ kg^−1^)	0.321	0.066	0.155	0.047
23	per animal (mg s^−1^)	12.343	0.565	3.566	1.242
	per weight (mg s^−1^ kg^−1^)	0.751	0.033	0.229	0.078
CYCLE	per animal (mg s^−1^)	12.343	0.103	2.210	1.093
	per weight (mg s^−1^ kg^−1^)	0.751	0.015	0.195	0.074

**Table 5:** Statistics for the evaluation of the performance of the models: root mean square error (*RMSE*), correlation coefficient (*r*), and mean absolute relative error (*MARE*).

Prediction Period	*RMSE*	*MARE*	*r*
60 min	1.21	0.23	0.63
10 min	1.27	0.25	0.56

## Data Availability

https://nubeusc-my.sharepoint.com/:x:/g/personal/manuelramiro_rodriguez_usc_es/EYK2Huu-QKNKjr7Tw3kJArIBEARe8WB1ONbMju4DjYsm0A?e=gS5bD4 (accessed on 30 March 2022).
